# Mental Health Among Medical Professionals During the COVID-19 Pandemic in Eight European Countries: Cross-sectional Survey Study

**DOI:** 10.2196/24983

**Published:** 2021-01-18

**Authors:** Svenja Hummel, Neele Oetjen, Junfeng Du, Elisabetta Posenato, Rosa Maria Resende de Almeida, Raquel Losada, Oscar Ribeiro, Vincenza Frisardi, Louise Hopper, Asarnusch Rashid, Habib Nasser, Alexandra König, Gottfried Rudofsky, Steffi Weidt, Ali Zafar, Nadine Gronewold, Gwendolyn Mayer, Jobst-Hendrik Schultz

**Affiliations:** 1 Department of General Internal and Psychosomatic Medicine Heidelberg University Hospital Heidelberg Germany; 2 Liyuan Hospital Tongji Medical College Huazhong University of Science and Technology Wuhan China; 3 Intras Foundation Valladolid Spain; 4 Center for Health Technology and Services Research Department of Education and Psychology University of Aveiro Aveiro Portugal; 5 Geriatric and NeuroRehabilitation Department AUSL-IRCCS Reggio Emilia Emilia Italy; 6 School of Psychology Faculty of Science and Health Dublin City University Dublin Ireland; 7 ZTM Bad Kissingen GmbH Bad Kissingen Germany; 8 R&D Department RDIUP Les Mureaux France; 9 Memory Clinic Institut Claude Pompidou Nice France; 10 Clinic for endocrinology and metabolic disorders Kantonspital Olten Olten Switzerland; 11 Department of Psychiatry, Psychotherapy and Psychosomatics Psychiatric Hospital Zurich University of Zurich Zurich Switzerland

**Keywords:** mental health, COVID-19, Europe, medical professionals, stress, depression, anxiety, coping, stressors

## Abstract

**Background:**

The death toll of COVID-19 topped 170,000 in Europe by the end of May 2020. COVID-19 has caused an immense psychological burden on the population, especially among doctors and nurses who are faced with high infection risks and increased workload.

**Objective:**

The aim of this study was to compare the mental health of medical professionals with nonmedical professionals in different European countries during the COVID-19 pandemic. We hypothesized that medical professionals, particularly those exposed to COVID-19 at work, would have higher levels of depression, anxiety, and stress. We also aimed to determine their main stressors and most frequently used coping strategies during the crisis.

**Methods:**

A cross-sectional online survey was conducted during peak COVID-19 months in 8 European countries. The questionnaire included demographic data and inquired whether the participants were exposed to COVID-19 at work or not. Mental health was assessed via the Depression Anxiety Stress Scales32 (23.53)–21 (DASS-21). A 12-item checklist on preferred coping strategies and another 23-item questionnaire on major stressors were completed by medical professionals.

**Results:**

The sample (N=609) consisted of 189 doctors, 165 nurses, and 255 nonmedical professionals. Participants from France and the United Kingdom reported experiencing severe/extremely severe depression, anxiety, and stress more often compared to those from the other countries. Nonmedical professionals had significantly higher scores for depression and anxiety. Among medical professionals, no significant link was reported between direct contact with patients with COVID-19 at work and anxiety, depression, or stress. “Uncertainty about when the epidemic will be under control” caused the most amount of stress for health care professionals while “taking protective measures” was the most frequently used coping strategy among all participants.

**Conclusions:**

COVID-19 poses a major challenge to the mental health of working professionals as a considerable proportion of our participants showed high values for depression, anxiety, and stress. Even though medical professionals exhibited less mental stress than nonmedical professionals, sufficient help should be offered to all occupational groups with an emphasis on effective coping strategies.

## Introduction

### Background

The death toll of COVID-19 topped 170,000 in Europe by the end of May 2020 [1]. Italy, Spain, France and the United Kingdom were among the worst affected countries with respect to high infection rates and overburdened health care systems [2-4]. Deaths of approximately 0.9 per 1 million inhabitants for Spain and France, 1.45 for Italy, and 3.94 for the United Kingdom were reported at the end of May. The death rate in Portugal was high as well with 1.32 deaths per million inhabitants [5]. Nevertheless, it was not one of the most affected countries [6,7]. Mortality in Germany was 0.47 deaths per 1 million inhabitants, Austria 0.52, and Switzerland 0.3 [5]; these values are lower but nevertheless alarming. As the number of cases soared, national governments introduced widespread restrictions to control the virus spread such as closing down borders, social distancing, travel restrictions, wearing of masks, working from home, and closure of public facilities [8,9]. The impending risk of infection, increasing number of COVID-19 cases, and the overburdening of health care systems created an unprecedented situation, which impacted not only everyday life but also the psychological welfare of the general population.

### COVID-19 and Mental Health

A recent study in 41 countries showed high stress levels in the general population during the COVID-19 pandemic, similar to those reported during the severe acute respiratory syndrome (SARS) epidemic in 2003 [10]. Depressive symptoms increased from 8.5% before COVID-19 to 27.8% during COVID-19 in a US study [11]. Studies have shown a similar picture for Europe. An Italian study concluded that leaving home for work was associated with increased stress, which may be due to the fear of getting infected [12]. In a study from Spain during the lockdown, about 34% and 21% of the participants reported moderate to extremely severe depression and anxiety, respectively [13]. A study from the United Kingdom reported high mental distress during the lockdown [14]. The first part of a French study in March 2020 found that the prevalence of anxiety among the general population was twice as high as reported in a study before COVID-19 [15]. In comparison, a study in Hong Kong found that about one-third of SARS survivors still suffered from moderate to severe or severe anxiety and/or depressive symptoms 4 weeks after their recovery [16]. Even 30 months after exposure to SARS, psychiatric disorders were prevalent among SARS survivors [17].

### COVID-19 and Medical Professionals

“Breathe. It’s what we all want these days, doctors and patients, nurses and care workers. All of us. We want air,” wrote an Italian frontline doctor from Milan in April during the peak of COVID-19 while sharing his routine of wearing a mask all the time [18].

Among the health care workers struggling to cope with the situation of doing their jobs while trying to protect themselves and their families [19], one would suspect doctors and nurses to be the most affected psychologically. During SARS, physicians who had direct contact with infected patients expressed greater mental distress, more stigmatization, and more worries about infecting their family [20]. This was confirmed during the COVID-19 pandemic in Italy where frontline health care workers reported posttraumatic stress symptoms [21]. Thousands of medical professionals were sent into quarantine after contracting COVID-19 in Italy, France, and Spain [22]. The fact that they came so close to the disease put their mental health at a higher risk than the general population [19]. The psychological challenges of the pandemic have been described for nurses and doctors in Europe [23,24]. Stronger effects on the mental health of medical health workers compared to nonmedical health workers [25] and a high prevalence of mental health symptoms among physicians and other medical staff have also been reported in China [26,27].

### Coping Strategies and Major Stressors

A 2012 study with nurses showed that negative coping strategies led to higher levels of mental distress whereas positive coping was partly negatively correlated with depression and anxiety levels [28]. During SARS, physicians in Toronto were mainly concerned about disparities in the health care of non-SARS patients because of the special situation [20] while nurses in Taiwan experienced “endangering of colleagues” as a major stressor [29]. Getting information about the virus and sticking to infection control procedures seemed to be important coping strategies [20,29]. During the COVID-19 pandemic, coping strategies and stressors of health care workers have been investigated in China, showing that health care workers worried a lot about possibly infecting their families and were highly stressed by witnessing the deaths of infected patients. Adhering to protective measures and learning more about COVID-19 were most often used to cope [30,31].

### Objectives

At a time when public health systems are overburdened in the fight against COVID-19 [2], physically and mentally healthy professionals are essential for the provision of reliable and efficient health care services. Physician burnout has been linked with medical errors [32] and further harmful effects for coworkers, patients, and the whole health care system [33]. The influence of COVID-19 on mental health in individual European countries and/or in individual population groups has been assessed, but a clear answer to the question of an overall impression of mental health in Europe during the peak months of the pandemic is still lacking. As global rates of infections rise once again and an effective vaccine remains unavailable, this question gains further relevance for determining mental health care needs for working professionals in the near future.

This study aimed to explore medical and nonmedical professionals’ mental health in different European countries during the 3-month state of emergency due to the pandemic and whether or not it was influenced by exposure to the virus at work. We hypothesized that medical professionals, particularly those exposed to COVID-19 at work, would show higher scores in depression, anxiety, and stress compared to nonmedical professionals. Moreover, we investigated which aspects of the COVID-19 pandemic worried medical professionals the most while at work and which coping strategies they most frequently employed. By uncovering these stressors and coping strategies, it might be possible to devise policies to prepare and support medical professionals better for future crises.

## Methods

### Study Design

We used a cross-sectional, multilingual survey design to investigate the mental health of working professionals in 8 European countries (Germany, the United Kingdom, Spain, France, Portugal, Austria, Italy, and Switzerland) during 3 months of the COVID-19 crisis between April 1 and June 20, 2020. The focus was on medical professionals and whether they were exposed to COVID-19 at work. Additionally, we asked about the most stressful aspects of work and coping strategies most often used*.* Ethical approval for this study was granted by the Ethics Commission of the Medical Faculty of Heidelberg University (S-361/2020).

### Participants

The participants were recruited online via public social networking groups and via the authors’ European contacts with partner organizations from international joint projects.

The sample (N=609) included 354 people with medical professions, including 189 doctors and 165 nurses (including geriatric care), and 255 people with nonmedical occupations (eg, teachers, office staff, psychologists, retired persons, social workers). Participants were aged 18-84 years (median 41 years) with 151 males and 458 females.

The percentage distribution of participants and professional groups in different European countries is summarized in Table 1.

**Table 1:** Distribution of professional groups within European countries.

Country	Medical professionals, n (%)	Nonmedical professionals, n (%)	Total, n
Germany	100 (73.53)	36 (26.47)	136
Austria	6 (28.57)	15 (71.43)	21
Switzerland	33 (82.50)	7 (17.50)	40
France	15 (28.85)	37 (71.15)	52
Italy	142 (89.31)	17 (10.69)	159
Spain	28 (28.28)	71 (71.72)	99
Portugal	25 (54.35)	21 (45.65)	46
United Kingdom	5 (8.93)	51 (91.07)	56

### Measurements

The survey consisted of a questionnaire derived from several validated instruments, with added items on demographics (eg, gender, age, marital status, etc) and a question on whether or not the participants were exposed to patients with COVID-19 at work.

Mental stress was assessed via the Depression Anxiety Stress Scales–21 (DASS-21)—a shorter version of the DASS-42 [34]—which is available in different languages. The DASS-21 consists of 21 items, which can be divided into 3 subscales, each containing 7 items to measure depression (eg, “I couldn't seem to experience any positive feeling at all”), anxiety (eg, “I was aware of dryness of my mouth”), and stress (eg, “I found it difficult to relax”). The responses are rated on a 4-point Likert scale ranging from 0 (did not apply to me at all) to 3 (applied to me very much or most of the time) to indicate how much the statement applied to the participant over the past week. According to Lovibond and Lovibond [34], the authors of the questionnaire, scores for the subscales are calculated by adding the answers of the 7 items for each subscale and then multiplying the result by 2 to get the total score for each participant for comparison to the DASS normative data [35].

To determine the most important stressors for medical staff, we used a questionnaire similar to the one used in a study by Lee et al [29] on SARS, which contains specific items for medical staff. The questionnaire consisted of 23 items. On a Likert scale from 0 (not at all) to 4 (very much), the participants indicated how often they thought about or were concerned about the individual stressors in their everyday life or at work. Given the focus of the study, this questionnaire was completed only by the medical staff participants.

Also based on Lee et al [29], we derived a questionnaire on coping strategies where participants could respond to 12 items using a scale from 0 (almost never) to 3 (almost always). Although all participants completed the coping strategies questionnaires, only results from the medical staff participants are presented here considering the aims and objectives of the study.

### Procedure

All questionnaires were translated from English by native speakers or professional translators for use in the respective countries. The survey was made available online via the Soscisurvey.de [36] platform. Consent to participate was obtained online. The English and German questionnaires were distributed at the beginning of April 2020, followed shortly after by the Italian version. The surveys in Spain and Portugal were launched in mid-April and in France in mid-May.

### Data Analysis

Using the Lovibond and Lovibond [34] method, the depression, anxiety, and stress subscales on DASS-21 were, according to individual sum scores, categorized as normal, mild, moderate, severe, and extremely severe [34]. These subscales were then grouped as normal/mild, moderate, and severe/very severe.

We created two groups: medical professionals consisting of doctors and nurses; and nonmedical professionals, which included other jobs in health care, volunteers, nonmedical staff, and community health care workers.

Descriptive analysis (including means, SDs, and frequencies) and inference statistics (multivariate analysis of variance [MANOVA]) were calculated using SPSS, version 24 (IBM Corp) [37].

## Results

### Distress Levels Across Surveyed European Countries

Across all surveyed countries, 65% (n=396) of the participants reported a normal/mild level of depression, followed by 18% (n=108) with moderate and 17% (n=105) with severe/extremely severe depression. Regarding anxiety, 63% (n=386) reported a normal/mild level of anxiety, 15% (n=91) a moderate level, and 22% (n=132) a severe/extremely severe level. In terms of stress, 59% (n=356) reported a normal/mild level, 14% (n=87) a moderate level, and 27% (n=166) a severe/extremely severe level. Tables 2-4 shows the mean scores for depression, anxiety, and stress for each of the 8 European countries as well as the percentage of participants assigned to the groups normal/mild, moderate, and severe/extremely severe.

**Table 2:** Depression levels in different European countries assessed using the Depression Anxiety Stress Scales–21.

Country	Mean (SD)^a^	Normal/mild, n (%)	Moderate, n (%)	Severe/very severe, n (%)
Germany	11.49 (8.91)	87 (63.97)	28 (20.59)	21 (15.44)
Austria	7.33 (8.23)	17 (80.95)	3 (14.29)	1 (4.76)
Switzerland	7.45 (8.68)	31 (7*7*.50)	6 (15.00)	3 (7.50)
France	17.42 (11.63)	22 (42.31)	11 (21.15)	19 (36.54)
Italy	10.03 (9.30)	119 (74.84)	19 (11.95)	21 (13.21)
Spain	8.51 (8.98)	75 (75.76)	14 (14.14)	10 (10.10)
Portugal	12.26 (8.46)	24 (52.17)	14 (30.44)	8 (17.39)
United Kingdom	17.64 (11.04)	21 (37.50)	13 (23.21)	22 (39.29)
Total	11.34 (9.90)	396 (65.02)	108 (17.73)	105 (17.24)

**Table 3:** Anxiety levels in different European countries assessed using the Depression Anxiety Stress Scales–21.

Country	Mean (SD)	Normal/mild, n (%)	Moderate, n (%)	Severe/very severe, n (%)
Germany	8.44 (7.94)	85 (62.50)	19 (13.97)	32 (23.53)
Austria	4.86 (5.68)	15 (71.43)	5 (23.81)	1 (4.76)
Switzerland	4.10 (6.13)	34 (85.00)	3 (7.50)	3 (7.50)
France	11.39 (10.53)	27 (51.92)	10 (19.23)	15 (28.85)
Italy	7.64 (8.39)	110 (69.18)	25 (15.72)	24 (15.09)
Spain	10.04 (10.54)	60 (60.61)	12 (12.12)	27 (27.27)
Portugal	9.83 (8.59)	23 (50.00)	11 (23.91)	12 (26.09)
United Kingdom	10.36 (9.69)	32 (57.14)	6 (10.71)	18 (32.14)
Total	8.61 (9.00)	386 (63.38)	91 (14.94)	132 (21.67)

**Table 4:** Stress levels in different European countries assessed using the Depression Anxiety Stress Scales–21.

Country	Mean (SD)	Normal/mild, n (%)	Moderate, n (%)	Severe/very severe, n (%)
Germany	17.13 (9.94)	76 (55.88)	27 (19.85)	33 (24.27)
Austria	14.10 (7.96)	17 (80.95)	1 (4.76)	3 (14.29)
Switzerland	11.40 (11.29)	32 (80.00)	2 (5.00)	6 (15.00)
France	21.77 (12.24)	25 (48.08)	4 (7.69)	23 (44.23)
Italy	17.25 (10.46)	91 (57.23)	27 (16.98)	41 (25.79)
Spain	16.42 (10.45)	62 (62.63)	13 (13.13)	24 (24.24)
Portugal	20.78 (10.95)	24 (52.17)	3 (6.52)	19 (41.30)
United Kingdom	18.86 (10.13)	29 (51.79)	10 (17.86)	17 (30.36)
Total	17.40 (10.71)	356 (58.46)	87 (14.29)	166 (27.26)

### Comparison of Medical With Nonmedical Professionals

A one-way MANOVA showed a significant main effect for profession (*F*_3,605_=5.019, *P*=.002, Wilk’s Λ=0.976). The effects were significant for depression (*F*_1,607_=7.929, *P*=.005) and anxiety (*F*_1,607_=5.87, *P*=.02], which indicated that medical professionals were less depressed (mean 10.39, SD 9.12) compared to nonmedical staff (mean 12.67, SD 10.77), as well as less anxious (mean 7.90, SD 8.36) than nonmedical staff (mean 9.65, SD 9.66). No statistically significant differences were found between medical professionals who had or had no exposure to COVID-19 at work (*F*_3,350_=0.525, *P*=.67, Wilk’s Λ=0.996).

Table 5 shows the 3 subscales of the DASS-21 and the percentage of medical and nonmedical professionals categorized as normal/mild, moderate, severe/extremely severe for each of these subscales.

**Table 5:** Overview of depression, anxiety, and stress levels for medical (n=345) and nonmedical professionals (n=255) assessed using the Depression Anxiety Stress Scales–21.

Participants		Mean (SD)	Normal/mild, n (%)	Moderate, n (%)	Severe/very severe, n (%)
**Depression**					
	Medical professionals	10.39 (9.12)	246 (69.49)	60 (16.95)	48 (13.56)
	Nonmedical professionals	12.67 (10.77)	150 (58.82)	48 (18.82)	57 (22.35)
	Total	11.34 (9.90)	396 (65.03)	108 (17.73)	105 (17.24)
**Anxiety**					
	Medical professionals	7.90 (8.36)	240 (67.80)	49 (13.84)	65 (18.36)
	Nonmedical professionals	9.65 (9.66)	146 (57.26)	42 (16.47)	67 (26.28)
	Total	8.61 (9.00)	386 (63.38)	91 (14.94)	132 (21.68)
**Stress**					
	Medical professionals	17.10 (10.51)	208 (58.76)	55 (15.54)	91 (25.71)
	Nonmedical professionals	17.80 (10.98)	148 (58.04)	32 (12.55)	75 (29.41)
	Total	17.40 (10.71)	356 (58.46)	87 (14.29)	166 (27.26)

### Stress Factors for Medical Professionals

The highest rated stressors were “uncertainty about when the epidemic will be under control” (mean 2.27, SD 0.85), “worry about inflicting COVID-19 on family” (mean 2.25, SD 0.99), “worry about nosocomial spread” (mean 2.02, SD 0.92) and a “frequent modification of infection control procedures” (mean 2.02, SD 0.89). Participants were least concerned about themselves (mean 1.12, SD 1.04) or coworkers (mean 1.25, SD 0.97) showing COVID-19–like symptoms, conflicts at work as the equivocal definition of responsibility between doctors and nurses (mean 1.19, SD 1.04), and blame from their commanding officers (mean 0.70, SD 0.95). An overview of all stressors in the order of reported severity can be found in Table 6.

**Table 6:** Stressors for doctors and nurses during COVID-19.

Items^a^	Responses, n	Mean (SD)
Uncertainty about when the epidemic will be under control	350	2.27 (0.85)
Worry about inflicting COVID-19 on family	351	2.25 (0.99)
Worry about nosocomial spread	348	2.02 (0.92)
Frequent modification of infection control procedures	350	2.02 (0.89)
Protective gears cause physical discomfort	349	1.75 (1.02)
Deterioration of patients’ condition	347	1.70 (1.00)
Worry about lack of proper knowledge and equipment	349	1.67 (1.04)
Worry about being negligent and endangering patients	350	1.66 (1.07)
Worry about getting infected	349	1.62 (1.03)
Patients’ emotional reaction	348	1.57 (0.96)
Worry about lack of manpower	348	1.56 (1.05)
Unclear documentation and reporting procedures	347	1.54 (1.01)
Patient families’ emotional reaction	346	1.52 (1.01)
Coworkers being emotionally unstable	348	1.52 (0.97)
Being without a properly fitted environment	348	1.51 (1.08)
Conflict between duty and safety	348	1.49 (1.07)
Worry about being negligent and endangering coworkers	351	1.48 (1.03)
Be infected by colleagues	349	1.31 (1.02)
Protective gear being a hindrance to providing quality care	349	1.28 (1.05)
Coworkers displaying COVID-19–like symptoms	347	1.25 (0.97)
Equivocal definition of the responsibility between doctors and nurses	346	1.19 (1.04)
Yourself displaying COVID-19–like symptoms	347	1.12 (1.04)
Blame from commanding officers	345	0.70 (0.95)

^a^Responses to the question: “When you think about COVID-19 in your life and work, how often did you think or worry about the following things?” (0=not at all, 3=very much).

### Coping Strategies of Medical Professionals

The most frequently used strategies were “taking protective measures” (mean 2.70, SD 0.57) and “actively acquiring more knowledge about COVID-19” (mean 2.34, SD 0.80). Alcohol and drugs were the least used strategy (mean 0.32, SD 0.60). Table 7 summarizes these results.

**Table 7:** Doctors’ and nurses’ coping strategies during COVID-19 (n=354).

Items^a^	Mean (SD)
Taking protective measures (washing hands, wearing a mask, taking own temperature, etc)	2.7 (0.57)
Actively acquiring more knowledge about COVID-19 (symptoms, transmission pathway, etc)	2.34 (0.80)
Video-chatting with family and friends by phone to share concerns and support	1.84 (0.87)
Engaging in recreational activities (online shopping, social media, internet surfing, etc)	1.62 (0.94)
Engaging in health-promoting behaviors (more rest, exercise, balanced diet, etc)	1.55 (0.99)
Switching thoughts and facing the situations with a positive attitude	1.54 (0.89)
Limiting oneself from watching too much news about COVID-19	1.37 (0.96)
Distracting oneself from thinking about COVID-19 issues by suppression or keeping busy	1.30 (0.92)
Acquiring mental health knowledge and information	1.01 (0.95)
Venting emotions by crying, screaming, smashing things, etc	0.50 (0.81)
Practicing relaxation methods (meditation, yoga, tai chi, etc)	0.46 (0.82)
Using alcohol or drugs	0.32 (0.60)

^a^Responses to the question: “When you think about COVID-19 in your life and work, how often did you use or try to use the following methods to handle the situation?” (1=almost never, 4=almost always).

## Discussion

### Overview

This study focused on doctors and nurses who were and are facing exceptional physical and mental challenges during the COVID-19 pandemic. In order to gain a deeper understanding of their situation, we investigated the perceived burden of different stressors on medical professionals as well as their coping strategies. Additionally, a general overview of the experienced stress, anxiety, and depression in various European countries during peak months of the pandemic was presented.

### Mental Health of Medical Professionals

The majority of doctors and nurses reported a normal to mild level of psychological strain, but about one-third expressed a moderate to extremely severe level of distress. Mental distress is associated with patient safety and a higher probability of medical errors [32,33]. Considering that long-term effects such as posttraumatic stress disorders are not uncommon among this professional group [38] and that the COVID-19 infection rate may increase again, our results should be taken seriously.

However, surprisingly, the mean scores for depression and anxiety among health care professionals were significantly lower than among nonmedical professionals. Regarding the level of stress, there was no significant difference between the two groups. These results are encouraging in the sense that the medical professionals—although confronted with difficult challenges and risks [19,39,40]—seemed to be mentally well prepared to handle the pandemic situation. A possible explanation could be, however, that their medical background helped them to better understand and classify COVID-19–related information when compared to their nonmedical counterparts. When they could feel self-sufficient, the situation appeared more manageable for them. A study with SARS survivors concluded that a better sense of self-care and self-efficacy led to better psychological adjustment to the situation [41]. In accordance with our results, in a study from Singapore, nonmedical health care professionals had a higher prevalence of anxiety than medical health care professionals during COVID-19. The authors believed that nonmedical health care professionals might have had less access to psychological support, less direct information about the situation and received less training on personal protective equipment and infection control measures [42]. In addition, previous European studies show that there is also an increased psychological burden during COVID-19 in the general population. For example, female gender and younger age were identified as risk factors [43,44], which were represented in large numbers in our study population. It has therefore already been recommended to take care of the mental health of the general population as well as special population groups [44].

Interestingly, we did not find any significant association between direct contact with COVID-19–infected patients at work and scores for anxiety, depression, or stress among medical professionals despite a previous study with health workers during the pandemic reporting more psychological distress when there was direct exposure to infected patients [21]. Not only in medical departments specializing in COVID-19 but in all medical units, protective measures such as permanent wearing of face masks, bans on visits to hospitals and nursing homes, and stricter hygiene regulations were made obligatory for medical personnel in the surveyed countries. In addition, because of the considerable number of deaths of doctors and nurses [45], COVID-19 would have been perceived by medical professionals as a kind of ever-present threat and not only when in direct contact with infected patients.

### Major Stressors and Coping Strategies

“Uncertainty about when the epidemic will be under control” and “worry about inflicting COVID-19 on family” were at the top of the list when medical professionals were asked about the most stressful things in their everyday life or at work during the pandemic. Possible infection of family is a major concern that has been reported several times before, for example, in the United Kingdom [38] or formerly among Taiwanese nurses during the SARS outbreak [29] and Chinese health care workers during COVID-19 [30]. Our results confirm the dilemma already mentioned by Perrin et al [19] during SARS: health care workers do their job by helping others but at the same time feel anxious about getting infected or infecting their families. Our participants were less worried about getting infected themselves than infecting their families with COVID-19.

The strategies most frequently used by medical professionals to deal with this unusual situation were “taking protective measures (washing hands, wearing a mask, taking own temperature)” and “actively acquiring more knowledge about COVID-19 (symptoms, transmission pathway, etc).” Effective protective measures were also the most common coping strategy among Taiwanese nurses during SARS [29] and Chinese health care workers during COVID-19 [30]. Another important strategy was “video-chatting with family and friends to share concerns and support,” which apparently had a higher priority for the participants in our study when compared to the nurses in Taiwan (“chatting with family and friends by phone to share concerns and support”) during SARS [29]. However, nowadays there are more possibilities, especially via social media, to be in touch digitally with friends and family compared to during the SARS outbreak. This has the advantage to get in touch directly with people experiencing mental burden, with the help of so-called e-mental health applications. The increasing role of these web-based interventions during the pandemic has already been observed [46]. While the acceptance of this development, especially among medical professionals, is high [47], different generations follow their own patterns of usage. However, all generations seek to stay related to their family members [48].

### COVID-19 and Mental Health in Europe

Our results show that although the majority of respondents reported normal to mild levels of depression, anxiety, and stress, the mean overall level of mental strain experienced was up to 2x higher compared to the normative data means of the DASS-21 [49]. However, according to DASS guidelines, it should be noted here that there is no DASS-21 cut-off for clinical diagnostics [35].

Our results concur with earlier studies about COVID-19 that have reported elevated levels of psychological distress during the pandemic [12-15,50,51]. However, these studies report only about a particular European country, which makes it clear that COVID-19 has a negative effect on the psyche but neglects that there can be differences across countries. The descriptive cross-sectional overview of our study shows that there are differences among countries in the numbers of people belonging to the severe/extremely severe category for depression, anxiety, and stress.

Participants from the United Kingdom and France showed, on a descriptive level, the highest scores for depression, anxiety, and stress when compared to other countries. This may be because England and France were among the countries most affected by COVID-19 [2] with a case fatality rate of 19.2% for France and 14.7% for the United Kingdom by the end of May [5]. Similar to our study, a previous study in the United Kingdom found elevated scores for depression and anxiety during COVID-19 [51], and a study from France presented a considerable prevalence of anxiety 1 week after the start of the lockdown [15]. In Italy and Spain, even though the situation was worse, the participants in our study showed lower scores of psychological strain compared to France and the United Kingdom. One reason for their lower scores of depression, anxiety, and stress could be the high proportion of medical professionals in the Italian sample, whose overall mental health was significantly better than that of the nonmedical professionals. Another reason could be that the surveys started at different points of time in these countries and the peak of the pandemic was different for each country.

The lower levels of psychological distress among participants in Austria and Switzerland could be attributed to the countries’ relatively lower number of cases per 1 million people [5]. In Germany, which had a higher number of cases [5] but less psychological strain, the health care system seemed to be better prepared as this is the country with the highest number of critical care beds in Europe [2,52].

### Limitations

Although our findings support previous studies on the psychological burden of COVID-19, a few limitations should be considered. Links to the online survey were distributed via social media and via the personal and professional networks of the authors. Since the contact networks in the individual European countries were not equally strong and online distribution of a link was difficult to control, the number of participants for each country was different, leading to uneven distribution of professional groups per country.

Moreover, the surveys did not start simultaneously in all European countries and data could not be acquired when the COVID-19 outbreak peaked in each country. In addition, translating questionnaires into different languages always carries the risk that the individual translations are not completely identical. Since we also partially adapted the already translated versions of DASS-21 to our online format, this could have led to an additional language bias. Finally, the category “nonmedical professionals” was heterogeneous. Persons who worked in nonmedical sectors of the health care system were included in this category and might have been exposed to COVID-19.

### Implications

The COVID-19 pandemic has caused fundamental changes in the health care and non–health care sectors and has put considerable strain not only on medical but also on nonmedical professionals. A sizeable part of participants expressed moderate to extremely severe symptoms of depression, anxiety, and/or stress while nonmedical professionals seemed to be more burdened than their medical counterparts. Targeted and personalized mental health services are needed not only for medical professionals but also for other professional groups during pandemics. When developing these services, specific needs and fears should be taken into account. One approach could be to examine the reasons why the medical staff are better at handling the pandemic situation and using these results to develop or optimize mental health services for future pandemics. By providing the opportunity for medical professionals to carry out their own protective measures and by providing sufficient information about the virus, they might be able to better overcome such situations. Further research is needed to analyze the long-term consequences of the psychological strain of COVID-19 by using valid diagnostic tools and other research designs like longitudinal surveys or qualitative studies. In-depth interviews could provide additional valuable information on major stressors and coping strategies.

## Abbreviations

DASS-21: Depression Anxiety Stress Scales–21
MANOVA: multivariate analysis of variance
SARS: severe acute respiratory syndrome
